# Nanoscale millefeuilles produce iridescent bill ornaments in birds

**DOI:** 10.1093/pnasnexus/pgae138

**Published:** 2024-04-03

**Authors:** Michaël P J Nicolaï, Gerben Debruyn, Mieke Soenens, Matthew D Shawkey, Liliana D’Alba

**Affiliations:** Department of Biology, Evolution and Optics of Nanostructures Group, University of Ghent, Ledeganckstraat 35, 9000 Gent, Belgium; Department of Recent Vertebrates, Royal Belgian Institute of Natural Sciences, Vautierstraat 29, 1050 Brussels, Belgium; Museum of Comparative Zoology, Harvard University, Cambridge, MA 02138, USA; Department of Biology, Evolution and Optics of Nanostructures Group, University of Ghent, Ledeganckstraat 35, 9000 Gent, Belgium; Department of Biology, Evolution and Optics of Nanostructures Group, University of Ghent, Ledeganckstraat 35, 9000 Gent, Belgium; Department of Biology, Evolution and Optics of Nanostructures Group, University of Ghent, Ledeganckstraat 35, 9000 Gent, Belgium; Department of Biology, Evolution and Optics of Nanostructures Group, University of Ghent, Ledeganckstraat 35, 9000 Gent, Belgium; Naturalis Biodiversity Center, Darwinweg 2, 2333 CR Leiden, The Netherlands

**Keywords:** structural color, iridescence, communication, birds

## Abstract

Colors are well studied in bird plumage but not in other integumentary structures. In particular, iridescent colors from structures other than plumage are undescribed in birds. Here, we show that a multilayer of keratin and lipids is sufficient to produce the iridescent bill of *Spermophaga haematina*. Furthermore, that the male bill is presented to the female under different angles during display provides support for the hypothesis that iridescence evolved in response to sexual selection. This is the first report of an iridescent bill, and only the second instance of iridescence in birds in which melanosomes are not involved. Furthermore, an investigation of museum specimens of an additional 98 species, showed that this evolved once, possibly twice. These results are promising, as they suggest that birds utilize a wider array of physical phenomena to produce coloration and should further stimulate research on nonplumage integumentary colors.

## Introduction

Iridescence, angle dependent coloration, is widespread in nature and occurs in almost all major animal clades (1, 2). It is a form of structural coloration that results from the interaction of light with materials with different refractive indices at a nanometer scale. In birds, iridescence is commonly found in feathers (3). These iridescent feathers are among the most colorful integumentary structures in nature, and as such considerable research has focused on its evolutionary significance (4), communication functions (4), and underlying structural (5) and genetic mechanisms (6). Nonetheless, iridescence in bird bills remains unreported, and colors in bird beaks were attributed to pigment-based and noniridescent structural coloration, i.e. in penguins where photonic structures consisting of 2D crystal lattices produce colors that are highly UV reflective.

Iridescence in birds has only been reported in feathers, where it is almost exclusively produced by the organization of melanosomes into thin layers, with the exception of a few examples where iridescence is produced by periodic matrices of air and β-keratin within barbs (7). Nonetheless, in many invertebrates, multiple highly specialized structures including thin film reflectors, multilayer reflectors, or other (chitin-based) photonic structures evolved to produce iridescent colors (2, 8, 9). Here, using a combination of spectrophotometry, microscopy, optical simulations, and phylogenetics, we show that the iridescent bill of the Western Bluebill (*Spermophaga haematina*) is produced by a multilayer of keratin and potentially lipids that evolved out of an ancestral bill with less-organized, thicker keratin layers, most likely in response to sexual selection.

## Results and discussion

**Fig. 1.**
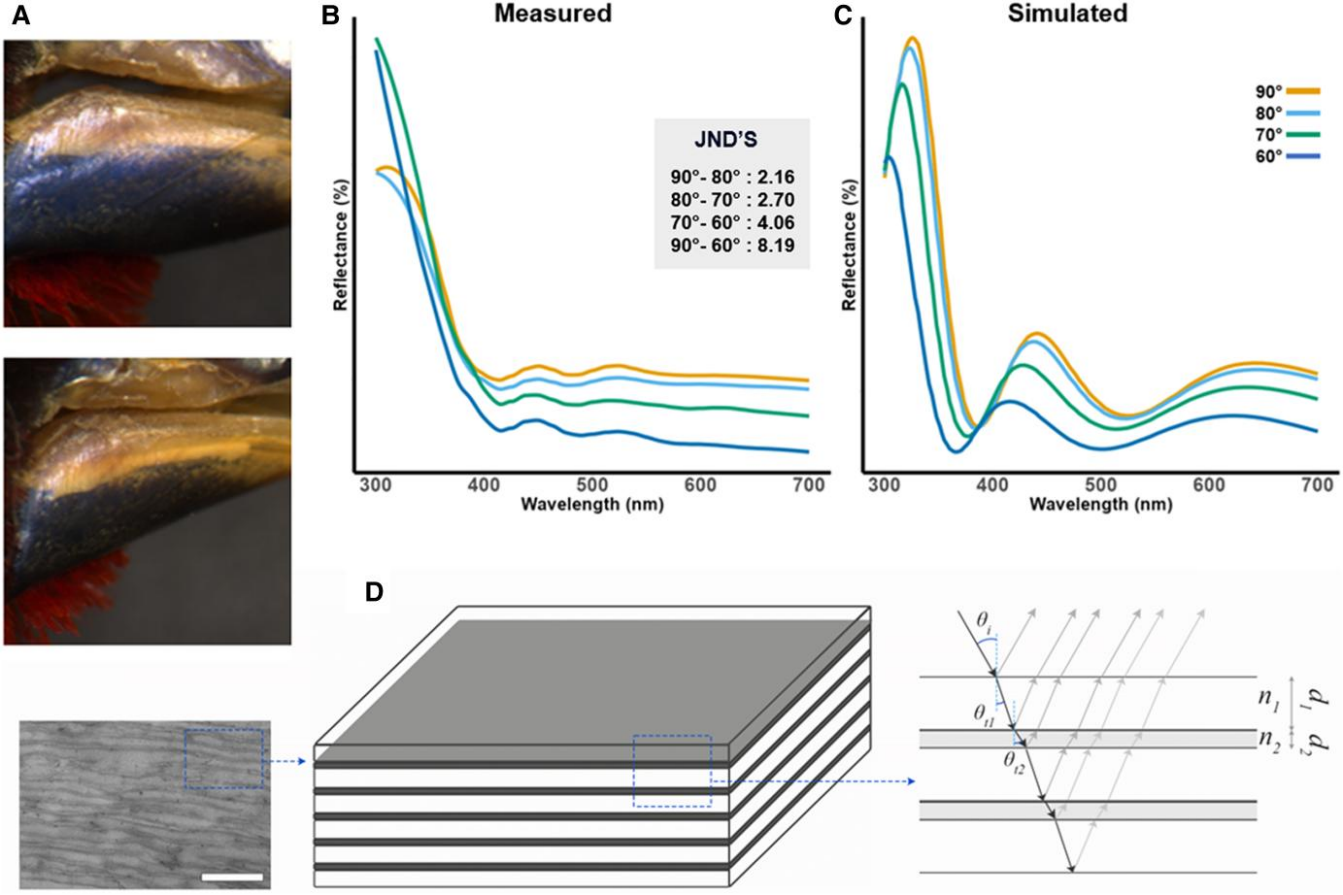
Western bluebill (*Spermophaga haematina*). A) Photographs of iridescent beak at different angles. B) Measured reflectance spectra at different angles with JND's. C) Modeled reflectance spectra at different angles resulting from finite-difference time-domain (FDTD) model. D) Simplified model illustrating the multilayer mechanism associated with the iridescent coloration.

Reflectance measurements (Supplementary data S1) showed that a distinct peak at the border between UV and blue wavelengths changes position, and intensity depending on the angle, confirming that the beak of *Spermophaga* is iridescent (Fig. 1A and B). Furthermore, modeling of the visual system of birds showed that chromatic differences between angles are perceivable (just noticeable difference [JND] >1).

**Fig. 2.**
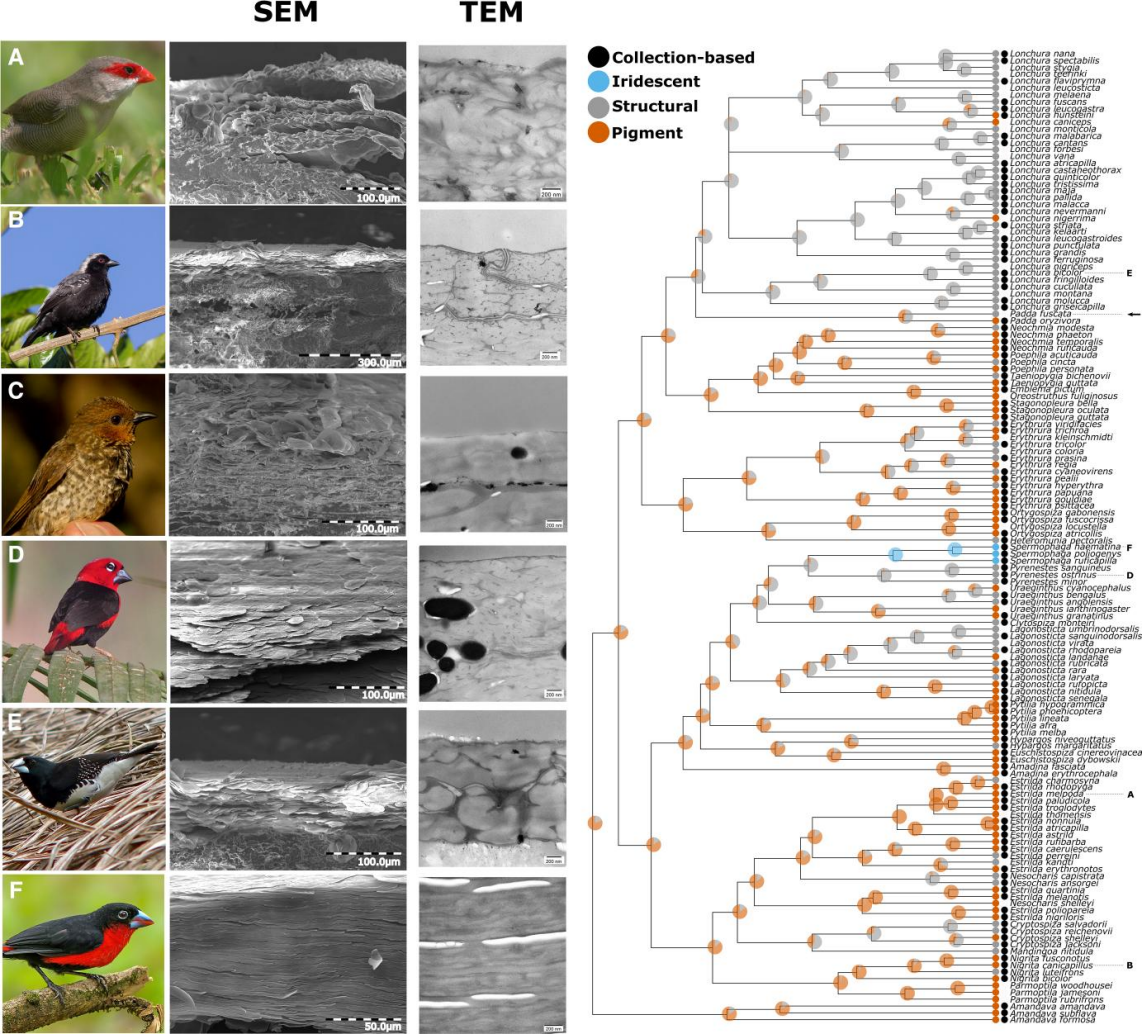
Overview figure showing the evolution of different color mechanisms (pigment-based = orange, containing a structural element = gray, iridescent = blue), together with six representative species with the SEM and TEM micrographs of their beaks. Species visually inspected using specimens (black dot) or using photographs (white dot) (Supplementary data S2 and S3). Pies represent probabilities of a color mechanism being present in ancestral nodes based on symmetrical evolutionary rates (i.e. the best model; Akaike information criterion values are 161.89 [equal rates], 138.23 [symmetrical rates], and 14354 [all rates different]) between color mechanisms. In general, evolution of color mechanisms is very conserved. Species used for SEM and TEM were *Estrilda melpoda* (A, pigment), *Nigrita canicapillus* (B, pigment), *Parmoptila woodhousei* (C, structural element), *Pyrenestes ostrinus* (D, structural element), *Spermestes bicolor* (E, structural element), and *Spermophaga haematina* (F, iridescent). *P. woodhousei* is not on the phylogenetic tree and its phylogenetic affinity is currently uncertain. The arrow corresponds to *Padda fuscata*, a potentially iridescent species.

Scanning electron microscopy (SEM) and transmission electron microscopy (TEM) imaging (Fig. 2) showed an increase in organization from pigment-based colors (Fig. 2A and B), via structure-based colors (Fig. 2C–E) to iridescence (Fig. 2F). This organization is hierarchical and visible at the micron scale (i.e. SEM images), and nanoscale (i.e. TEM images). This organization in layers is mostly present in the iridescent bill while absent in noniridescent bills. Furthermore, layers are significantly thinner and form a multilayer in the iridescent bill, essential for the production of iridescent colors (Fig. 1F). While unable to chemically quantify the composition of the different layers, multiple indications suggests that the beak consists of a keratin-lipid multilayer. First, the major component of a birds’ beak is keratin, produced in keratinocytes. These cells and their membranes are visible in the TEM images (Fig. 2). The layering consist of highly elongated cells, where the thickest layer of the multilayer is keratin, and the thinnest is formed by the cell membranes (lipids) of the keratinocytes that contain the keratin and other remnants of the cell (10). Finally, optical modeling based on TEM measurements confirmed that a multilayer consisting of five stacks of ultrathin keratin (thickness = 60 nm) alternating with a layer (thickness = 35 nm) with refractive index = 1.4 (i.e. lipids originating from the cell membranes of keratinocytes), was sufficient to produce the colors we observed, under all angles observed (Fig. 1C and D, Supplementary data S4 and S5). *Spermophaga haematina*'s iridescent colors are thus produced by a unique mechanism in birds, i.e. a series of keratin sheets parallel to the surface interchanged by cell membranes, forming a multilayer.

The iridescence observed in the bill *of S. haematina* differs from other iridescent colors that are produced by thin layers formed by melanosomes or barb nanomorphology (7). However, the use of lipids in multilayers to produce iridescent colors is known in plants (11). While melanin is present in most noniridescent bills, in the iridescent bill it is only present in the thicker, most basal layers. As such, it apparently has no function in color production other than perhaps to absorb incoherently scattered light (12). Such multilayer-based iridescence can also be found in manakins (7) and arthropods (9). Similar, but distinct, multilayer arrays were found in penguins, where they are formed by quasiparallel folded membrane doublets that have varying orientations with respect to the surface and produce UV colors (13).

While structural color evolved multiple times out of an ancestor with pigment-based bill coloration, the iridescent multilayer evolved only once, out of other structurally colored bills, evolving more but thinner multilayers, and suggesting that the presence of a structure is an evolutionary prerequisite for iridescence to evolve in the bill (Fig. 2). Interestingly, iridescent and noniridescent beaks with multilayers differ in the absence of melanosomes in the iridescent beak, likely a result of developmental constraints where melanosomes have larger dimensions than the thickness of the keratin layers necessary for iridescence. Given the many functions of melanin in beaks and other integuments (14), it is possible that selection against the loss of melanin might hinder the evolution of iridescent beaks. Nonetheless, a few estrildids (e.g. *Lonchura* sp. and *Padda fuscata*) might show weak iridescence, suggesting at least two origins (Fig. 2). Unfortunately these specimens were not present in the collection, warranting future additional research. Interestingly, in the clades that contain *Lonchura*/*Padda* and *Spermophaga*, weak iridescent plumage is present, suggesting that estrildids might have co-opted a bias for iridescent colors. To our knowledge, other families (e.g. Ploceidae and Vangidae) might have only noniridescent glossy or blue beaks.

Dense stacking and multilayers involving keratin are known to enhance mechanical strength (15). Hence, iridescence might have evolved as a byproduct of an adaptation for increased bill strength. Indeed, not all brightly colored integuments are ornamental: the signal has to be visible to the receiver, especially during courtship, and should elicit a response. Bills seem to fit the bill, as they are often sexually dimorphic, used in complex courtship rituals, associated with mating preference, indicate age, sex, social status, and fitness in both passerine and nonpasserine (reviewed in Ref. (16)). Interestingly, the bill of the western bluebill is sexually dichromatic, with males having more color. Furthermore, males of the genus *Spermophaga* use their iridescent bill during courtship, when they hold a leaf in the bill, mandibulate, and display the bill from different angles (shifting from −45° to 70°) by twisting its head (17). These results show that bluebills both perceive and use iridescence in courtship, suggesting a role for sexual selection. Indeed, bills as a whole are likely important sexual signals in Estrildids given the occurrence of sexual dichromatism in few species.

The presence of keratin-based iridescence raises the question why this color mechanism is so rare in birds (where iridescence is widespread) or almost absent in other vertebrates such as mammals (except for Chrysochloridae (18)) where this would drastically increase the attainable color space. One explanation might be that avian keratins are mostly composed of β-keratins (twisted β-sheet structures) opposed to the α-helical structure of mammalian α-keratin. Alternatively, the conditions for keratin-based iridescence to evolve might be rare and counterbalanced by other selective forces, or weak selection due to the restricted color vision in mammals. The presence of a keratin-cell membrane multilayer producing iridescence, highlights the need to investigate the function and color mechanisms of lesser studied integumentary structures (14, 16).

## Supplementary Material

pgae138_Supplementary_Data

## Data Availability

Data are available as supporting information. Code and data files can be found in the Dryad data repository 10.5061/dryad.ns1rn8q0x.

## References

[1] Sanders JV . 1964. Colour of precious opal. Nature. 204:1151–1153.

[2] Parker AR, McPhedran RC, Mckenzie DR, Botten LC, Nicorovici N-A. 2001. Photonic engineering. Aphrodite's iridescence. Nature. 409:36–37. 11343102 10.1038/35051168

[3] Auber L . 1957. The distribution of structural colours and unusual pigments in the Class Aves. Ibis. 99(3):463–476.

[4] Doucet SM, Meadows MG. 2009. Iridescence: a functional perspective. J R Soc Interface. 6:S115–S132. 19336344 10.1098/rsif.2008.0395.focus PMC2706478

[5] Nordén KK, Eliason CM, Stoddard MC. 2021. Evolution of brilliant iridescent feather nanostructures. eLife. 10:e71179. 34930526 10.7554/eLife.71179 PMC8691833

[6] Rubenstein DR, et al 2021. Feather gene expression elucidates the developmental basis of plumage iridescence in African starlings. J Hered. 112:417–429. 33885791 10.1093/jhered/esab014 PMC11502951

[7] Igic B, D’Alba L, Shawkey MD. 2016. Manakins can produce iridescent and bright feather colours without melanosomes. J Exp Biol. 219(12):1851–1859. 27307543 10.1242/jeb.137182

[8] Vukusic P, Sambles JR, Lawrence CR, Wootton RJ. 2001. Now you see it—now you don’t. Nature. 410:36. 10.1038/35065161 11242032

[9] Seago AE, Brady P, Vigneron J-P, Schultz TD. 2008. Gold bugs and beyond: a review of iridescence and structural colour mechanisms in beetles. J R Soc Interface. 6:S165–S184. 18957361 10.1098/rsif.2008.0354.focus PMC2586663

[10] Seki Y, Bodde SG, Meyers MA. 2010. Toucan and hornbill beaks: a comparative study. Acta Biomater. 6(2):331–343. 19699818 10.1016/j.actbio.2009.08.026

[11] Middleton R, et al 2020. Viburnum tinus fruits use lipids to produce metallic blue structural color. Curr Biol. 30(19):3804–3810. 32763166 10.1016/j.cub.2020.07.005

[12] Shawkey M, Hill GE. 2015. Carotenoids need structural colour to shine. Biol Lett. 1(2):121–124. 10.1098/rsbl.2004.0289 PMC1626226 17148144

[13] Dresp B, Jouventin P, Langley K. 2005. Ultraviolet reflecting photonic microstructures in the King Penguin beak. Biol Lett. 1(3):310–313. 17148195 10.1098/rsbl.2005.0322 PMC1617153

[14] Nicolaï MPJ, Shawkey MD, Porchetta S, Claus R, D’Alba L. 2020. Exposure to UV radiance predicts repeated evolution of concealed black skin in birds. Nat Commun. 11:2414. 32415098 10.1038/s41467-020-15894-6 PMC7229023

[15] Soons J, et al 2012. Multi-layered bird beaks: a finite-element approach towards the role of keratin in stress dissipation. J R Soc Interface. 9:1787–1796. 22337628 10.1098/rsif.2011.0910 PMC3385763

[16] Iverson ENK, Karubian J. 2017. The role of bare parts in avian signaling. Auk. 134(4):587–611.

[17] Payne RB . 2020. Red-headed bluebill (*Spermophaga ruficapilla*), version 1.0. In: del Hoyo J, Elliott A, Sargatal J, Christie DA, de Juana E, editors. Birds of the world. Ithaca (NY): Cornell Lab of Ornithology. 10.2173/bow.rehblu1.01

[18] Snyder HK, et al 2012. Iridescent colour production in hairs of blind golden moles (Chrysochloridae). Biol Lett. 8(3):393–396. 22279154 10.1098/rsbl.2011.1168 PMC3367760

