# Supplementary material for: Nanoscale millefeuilles produce iridescent bill ornaments in birds
Source: PNAS Nexus. 2024 Apr 3;3(4):pgae138. doi: 10.1093/pnasnexus/pgae138 (PMC11026107; doi:10.1093/pnasnexus/pgae138)
Supplement: pgae138_Supplementary_Data [file pgae138_supplementary_data.docx]

# **Material and methods**

## Reflectance measurements and visual modelling

Reflectance spectra were measured from one male specimen using AvaSpec-ULS2048 L StarLine Versatile Fibre-optic Spectrometer UV-VIS (300-700 nm) with an AvaLight-DH-S Deuterium-Halogen Light Source. We used a modified goniometer that rotates both the specimen and the probe to avoid shadows created by the curvature of the bill and measure colour at a single point of the beak. The light source was varied at eight different angles (from 55° to 90°) to measure changes in hue which are characteristic for iridescent colouration. We measured three different locations on the bill. Distance between probe and sample were kept equal between locations (Supplementary data 1). To verify whether measured colour differences (i.e. iridescence) is perceivable by birds, we calculated chromatic just-noticeable differences (JNDs) (Vorobyev & Osorio, 1998) between different angles using relative cone stimuli and an avian UV visual system obtained in ‘pavo’ (Maia et al., 2019). JNDs represent how colour is perceived by organisms, incorporating information of organismal visual systems. More specifically, differences with JND values below <1 are not perceivable to be different, whereas differences with JND > 1 are.

## Scanning- and Transmission Electron Microscopy

We used scanning- and transmission electron microscopy (SEM and TEM) to identify the mechanisms responsible for the iridescent bill and how histology differs from other non-iridescent birds. To do so we sampled six species with different colour mechanisms; *Estrilda melpoda* (carotenoid-based), *Nigrita canicapillus* (melanin-based), *Parmoptila woodhousei* (glossy, structural and melanin–based), *Pyrenestes ostrinus* (structural), *Spermestes bicolor* (structural) and *Spermophaga haematina* (iridescent). For TEM imaging, we embedded samples in Epon following standard protocols (Electron Microscopy Solutions), then cut thin (100-150 nm) sections using a Leica UC-6 microtome (Leica Microsystems, Germany). Thin sections were transferred to formvar-coated copper grids and stained with 1% uranyl acetate and lead citrate. Afterwards they were imaged using a JEOL JEM 1010 (JEOL Ltd, Japan) transmission electron microscope. For SEM imaging, we cut out a section of beak of each species and mounted it on an aluminium stub using carbon tape, and sputter-coated them with gold-palladium (10nm thick). Images were taken with a FlexSEM 1000 (Hitachi High-Tech Corporation, Japan) under an accelerating voltage of 5 -10 kV and working distances ranging from 6.6 to 7.5 mm. Measurements of the individual layers were measured using ImageJ (Schneider et al., 2012).

## Optical modelling

Optical simulations to verify the mechanism of iridescence were performed using the finite-difference time-domain (FDTD) modelling software (Ansys Lumerical Solutions). TEM images suggested that there were alternating light and dark bands which correspond to keratin and cell membranes with different refractive indices (RI) (similar to Lee et al., 2014) (Figure 2). Using the dimensions obtained from the TEM micrographs (63 nm for light, keratinous bands and 35 nm for suspected lipid membranes), we generated a multi-layered structure comprised of 10 layers of alternating keratin (RI = 1.55, Jeon et al., 2023) and lipids and possibly other organic remnants originating from the cell membranes of the keratinocytes (RI = 1.4, Ardhammar et al., 2002; Gadomski et al., 2019) in Lumerical. We applied a BFAST plane wave type and plane wave light source shape. BFAST plane wave overrides the boundary conditions along all axes enabling to obtain the reflected light from the UV-Visible wavelengths (300-700 nm) by the multi-layered structure at different angles of incidence (90° down to 60°, 10° steps).

## Phylogenetic analyses

To reconstruct the evolution of the iridescent bill, and to estimate the ancestral states of major colour mechanisms in Estrildids, we checked the presence of 1) distinct iridescent bills (i.e. as in *Spermophaga*), 2) other colours produced by structure rather than pigments (i.e. non-iridescent blue, white or glossy bills) or 3) colours produced by pigments (i.e. carotenoids and melanins) only. Combinations of structure and melanin pigments, such as glossy (rather than matte) melanin- or carotenoid-coloured bills, were scored as structural since this is the evolutionary unit that apparently changes. This choice was made based on TEM data that showed that the iridescent bill was functionally nested within structured, i.e. both structure and iridescence show layering (Figure 2). Initially, we examined 174 skins of 99 species of birds belonging to the Estrildidae family (70% of the species, all genera, supplementary data 2-3). Specimens originated from the natural history collection of the Royal Belgian Institute for Natural Sciences (RBINS), the Royal Museum for Central Africa (RMCA) and the Museum of Comparative Zoology (MCZ). We looked at all estrildid species present in these three collections. When specimens were available, we looked at one male and one female specimen. Additionally, we (M.P.J. and G.D) used descriptions and standardized drawings from Handbook of the Birds of the World (HBW) as well as images online to score all species independently (supplementary data 3). Scorers and methodology (image-based vs specimen based) yielded similar results (~90% match). None of these missing species (i.e. those that were only scored using images) showed any signs of iridescence, although few species might show weak iridescence (e.g. *Lonchura* sp. and *Padda fuscata*). Unfortunately, these species were not present in the collections visited for verification. We used the function “ace” of the ape package (Paradis et al., 2004) to estimate ancestral character states using three different models: all rates different (ARD), an all rates equal (ER) model and a symmetrical rates model (SYM). All phylogenetic analyses were run on the complete Bayesian maximum clade credibility species-level avian phylogeny from the Bird Tree Project (Jetz et al., 2012), built based on both genetic and taxonomic information and the higher-order relationship backbone from Hackett et al. (2008). Since species without genetic information have been placed in concordance with taxonomy, and since colour mechanisms seem to be evolutionary conserved, it is unlikely that using the genetic information-only tree will affect these analyses.

**References**

Ardhammar, M., Lincoln, P., & Nordén, B. (2002). Invisible liposomes: refractive index matching with sucrose enables flow dichroism assessment of peptide orientation in lipid vesicle membrane. *Proceedings of the National Academy of Sciences*, *99*(24), 15313–15317.

Birds of the World (2022). Edited by S. M. Billerman, B. K. Keeney, P. G. Rodewald, and T. S. Schulenberg. Cornell Laboratory of Ornithology, Ithaca, NY, USA. <https://birdsoftheworld.org/bow/home>

Gadomski, A., Kruszewska, N., & Rubi, J. M. (2019). Derivation of the refractive index of lipid monolayers at an air-water interface. *Optical Materials*, *93*, 1–5.

Hackett, S.J., Kimball, R.T., Reddy, S., Bowie, R.C., Braun, E.L., Braun, M.J., Chojnowski, J.L., Cox, W.A., Han, K.-L., Harshman, J., Huddleston, C.J., Marks, B.D., Miglia, K.J., Moore, W.S., Sheldon, F.H., Steadman, D.W., Witt, C.C., Yuri, T. (2008). A phylogenomic study of birds reveals their evolutionary history. Science, 320(5884), 1763-1768.

Jeon, D., Ji, S., Lee, E., Kang, J., Kim, J., D’Alba, L., Manceau, M., Shawkey, M.J., Yeo, J. (2023). How keratin cortex thickness affectsridescent feather colours. Royal Society Open Science, 10, 220786.

Jetz, W., Thomas, G.H., Joy, J.B., Hartmann, K., Mooers, A.O. Nature, 491, 444-448.

Lee, N., Horstemeyer, M.F., Rhee, H., Nabors, B., Liao, J., Willimans, L.N. (201). Hierarchical multiscale structure-property relationships of the red-bellied woodpecker (Melanerpes carolinus) beak. Journal of the Royal Society Interface, 11: 20140274.

Maia, R., Gruson, H., Endler, J.A., White, T.E. (2019). Pavo2: New tools for the spectral and spatial analysis of colour in R. Methods in Ecology and Evolution, 10(7), 1097-11°7.

Schneider, C.A., Rasband, W.S., Eliceiri, K.W. (2012). NIH Image to ImageJ: 25 years of image analysis. Nature Methods, 9(7), 671-675.

Vorobyev, M. and Osorio, D. (1998). Receptor noise as a determinant of colour thresholds. Proceedings of the Royal Society London B: Biological Sciences, 265(1394), 351–358. Fan, X., Zheng, X., An, T., Li, X., Leung, N., Zh B., Sui, T., Shi, N., Fan, T., Zhao, Q. (2023). Light diffraction by sarcomeres produces iridescence in transmission in the transparent ghost catfish. Proceeding of the National Academy of Sciences. 120(12), e2219300120.
